# IL-4-producing ILC2s are required for the differentiation of T_H_2 cells following *Heligmosomoides polygyrus* infection

**DOI:** 10.1038/mi.2016.4

**Published:** 2016-02-17

**Authors:** VS Pelly, Y Kannan, SM Coomes, LJ Entwistle, D Rückerl, B Seddon, AS MacDonald, A McKenzie, MS Wilson

**Affiliations:** 1 Mill Hill Laboratory, The Francis Crick Institute, London, UK; 2 Institute of Immunology and Infection Research (3IR), University of Edinburgh, Edinburgh, UK; 3 Institute of Immunity and Transplantation, UCL, London, UK; 4 Faculty of Life Sciences, University of Manchester, Manchester, UK; 5 MRC Laboratory of Molecular Biology, University of Cambridge, Cambridge, UK

## Abstract

Immunity to many human and murine gastrointestinal helminth parasites requires interleukin-4 (IL-4)-directed type 2 helper (T_H_2) differentiation of CD4^+^ T cells to elicit type-2 immunity. Despite a good understanding of the inflammatory cascade elicited following helminth infection, the initial source of IL-4 is unclear. Previous studies using the rat helminth parasite *Nippostronglyus brasiliensis*, identified an important role for basophil-derived IL-4 for T_H_2 differentiation. However, basophils are redundant for T_H_2 differentiation following infection with the natural helminth parasite of mice *Heligmosomoides polygyrus*, indicating that other sources of IL-4 are required. In this study using *H. polygyrus*, which is controlled by IL-4-dependent immunity, we identified that group-2 innate lymphoid cells (ILC2s) produced significant amounts of IL-4 and IL-2 following *H. polygyrus* infection. Leukotriene D4 was sufficient to stimulate IL-4 secretion by ILC2s, and the supernatant from activated ILC2s could potently drive T_H_2 differentiation *in vitro* in an IL-4-dependent manner. Furthermore, specific deletion of IL-4 from ILC2s compromised T_H_2 differentiation *in vivo*. Overall, this study highlights a previously unrecognized and important role for ILC2-derived IL-4 for T_H_2 differentiation in a natural T_H_2-dependent model of human helminthiasis.

## Introduction

Intestinal helminth infections remain a significant global burden with devastating economic and health impacts,^1^ highlighting clear gaps in our knowledge and translation of anti-helminth immunity. Most small animal model systems are not permissive hosts for human helminths, limiting our ability to study natural, coadapted host–pathogen interactions. Instead, natural murine helminths, such as *Heligmosomoides polygyrus*, which establish chronic infections in inbred mice similar to their human counterparts, provide an appropriate experimental system to study natural host–pathogen interactions.

Immunity to *H. polygyrus* has been shown to be dependent on CD4^+^ T cells and interleukin (IL-4)^2^ orchestrating a polarized type-2 immune response, activating and mobilizing a suite of innate immune cells and local tissue responses. Early innate responses can determine the outcome, severity, and persistence of infection;^3^ however, our understanding of these early events is incomplete. Specifically, IL-4-dependent differentiation of CD4^+^ Type 2 helper (T_H_2) cells is an essential component of immunity to *H. polygyrus*;^4^ however, the *in vivo* source of IL-4 remains elusive. A series of studies identified that basophils were an important source of IL-4 for T_H_2 differentiation during murine infection with the rat parasite *Nippostrongylus brasiliensis*.^5^ However, given that IL-4 is not required for immunity to *N. brasiliensis*,^6^ this experimental system may not be the most appropriate to identify the early sources of IL-4 during natural immunity. Basophils and eosinophils express and secrete IL-4 during *H. polygyrus* infection;^7^ however, T_H_2 differentiation and type-2 immunity was not impaired in basophil-deficient mice, basophil-depleted mice, or mice given anti-IL-5 to deplete eosinophils.^2^,^8^ Thus, although IL-4 is critical for T_H_2 differentiation during *H. polygyrus* infection, neither basophils nor eosinophils are essential sources of IL-4.

Group-2 innate lymphoid cells (ILC2s) have a variety of important functions including the secretion of potent type-2 cytokines IL-13, IL-5, and IL-9, which contribute to anti-helminth immunity as well as the pathogenesis of allergy.^9^,^10^ Recent evidence identified ILC2s as antigen-presenting cells able to process and present antigen to CD4^+^ T cells and relay signals to the adaptive immune system.^11^ ILC2s also contribute to the maintenance of other innate cells in the tissue,^12^,^13^ support tissue remodeling and repair following injury,^14^ and maintain metabolic homeostasis.^15^ ILC2s have been shown to transcribe^16^ and secrete IL-4;^17^ however, the functional relevance of ILC2-derived IL-4 has not been tested.

In this study, we report that ILC2s expand early during *H. polygyrus* infection in both the intestinal lamina propria (LP) and in the draining mesenteric lymph nodes (MLNs) in concert with early T_H_2 cell differentiation. ILC2 expansion with IL-2 immune complexes invoked a local type-2 response in the tissue and provided some protection from chronic *H. polygyrus* in the absence of *Rag*-dependent cells. Furthermore, LP ILC2s expressed *Il4* and *Il2* transcripts and secreted large amounts of IL-4 and IL-2 protein. Functionally, ILC2-derived IL-4 was required for the recruitment of ILC2s to the tissue following *H. polygyrus* infection and to drive optimal CD4^+^ T_H_2 cell differentiation. These data identify a previously overlooked and important role for ILC2-derived IL-4 for T_H_2 cell differentiation following *H. polygyrus* infection. Harnessing the influence of ILC2s may, therefore, support T-cell-mediated vaccine approaches against intestinal helminth infections.

## Results

### Development of type-2 responses following *H. polygyrus* infection correlates with the expansion of ILC2s in the local lymph node and SI

**Figure 1:**
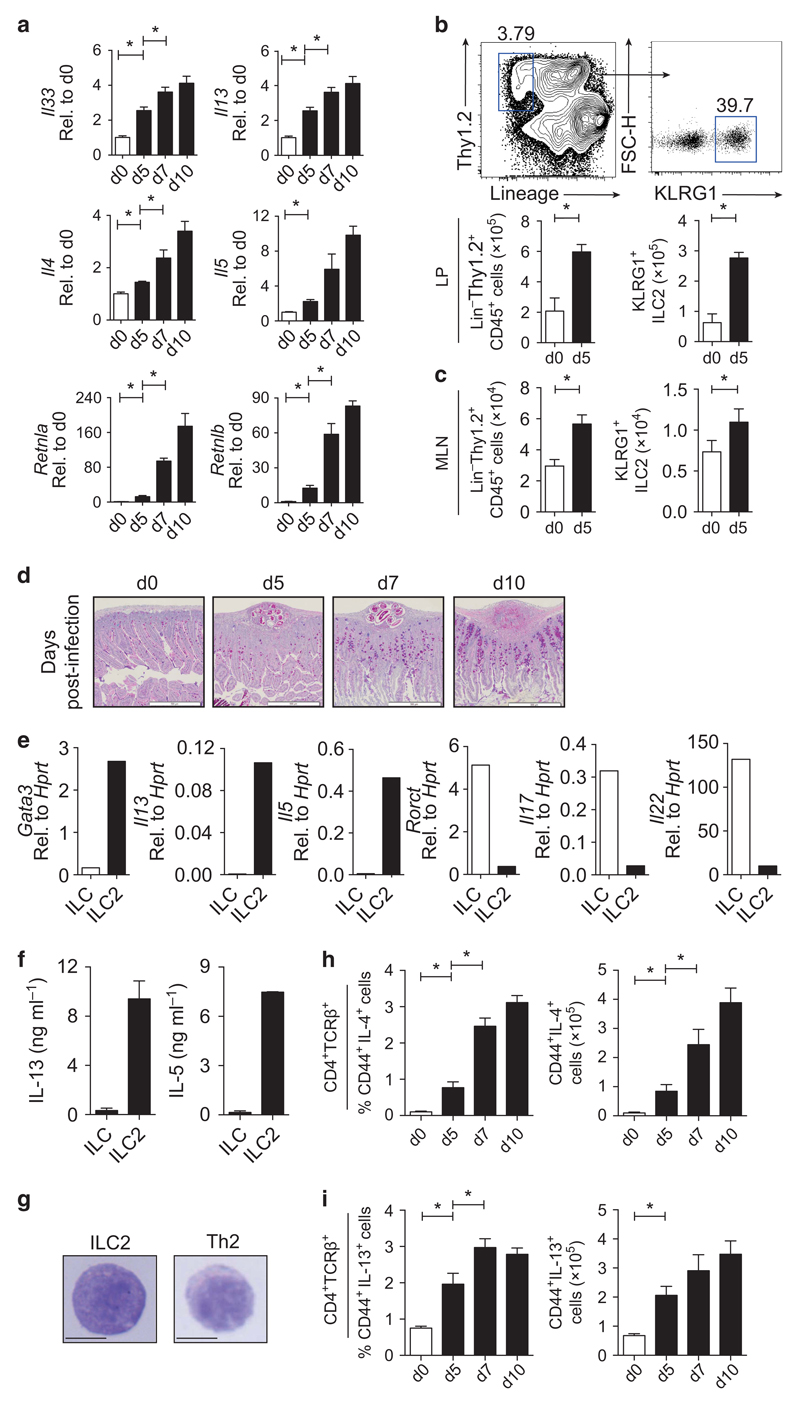
Development of type-2 responses to *Heligmosomoides polygyrus* correlates with the expansion of group-2 innate lymphoid cells (ILC2s) in the small intestine (SI). C57BL/6 mice were infected with 200 *H. polygyrus* larvae and harvested at days 0, 5, 7, and 10 postinfection. (**a**) Gene expression of *Il33*, *Il13*, *Il4*, *Il5*, *Retnla*, and *Retnlb* in the SI day 5 postinfection, expressed as fold change relative to day 0. (**b**) Representative fluorescence-activated cell sorting (FACS) plots of lamina propria (LP) KLRG1^+^ ILC2s within the Lin^−^Thy1.2^+^ gate. Total number of Lin^−^Thy1.2^+^ ILCs and Lin^−^Thy1.2^+^ KLRG1^+^ ILC2s in the (**b**) LP and (**c**) mesenteric lymph node (MLN). LP data are representative of three independent experiments with four mice per group. MLN data is pooled from three independent experiments with four mice per group. (**d**) Mucus staining (Alcian blue-periodic acid-Schiff) of SI sections of infected mice. *Rag2*^−/−^ mice were infected with 200 *H. polygyrus* larvae and harvested at day 5 postinfection. (**e**) Gene expression in sort-purified LP ILCs (Lin^−^Thy1.2^+^ KLRG1^−^) and ILC2s (Lin^−^Thy1.2^+^ KLRG1^+^) at day 5 postinfection, expressed relative to *Hprt*. (**f**) Interleukin-5 (IL-5) and IL-13 protein production by sort-purified ILCs and ILC2s stimulated with phorbol 12-myristate 13-acetate (PMA) and ionomycin (PMA + I) for 24 h. Cells were pooled from four to six mice, with error bars indicating technical replicates. (**g**) Giemsa-stained Lin^−^Thy1.2^+^ KLRG1^+^ ILC2s and CD4^+^ TCRβ^+^*Il4*-GFP^+^ cells (bar represents 100 μm). (**h**) Frequency and (**i**) total number of CD44^+^ IL-4^+^ cells and CD44^+^ IL-13^+^ CD4^+^ TCRβ^+^ cells in the spleen of C57BL/6 mice following *H. polygyrus* infection as assessed by intracellular cytokine staining. Data are representative of three independent experiments with four to six mice per group. *P≤0.05 using the Mann–Whitney test. FSC-H, forward side scatter of height; TCR, T-cell receptor.

ILC2s are recruited to local lymph nodes of mice early after infection with the natural murine helminth, *H. polygyrus*,^18^ with the total number of ILC2s correlating with resistance to *H. polygyrus*.^19^ However, the functional relevance of ILC2s in *H. polygyrus* infection has not been tested. Wild-type (WT) C57BL/6 mice infected with *H. polygyrus* had an elevated local type-2 response in the small intestine (SI) from day 5 following infection, with elevated expression of *Il33* and type-2 cytokines (*Il13*, *Il5*, and *Il4*) (Figure 1a). Anti-helminth type-2 innate effector pathways were also increased, including macrophage-associated *Retnla* and goblet cell-derived *Retnlb* (Figure 1a). Concomitant with this early type-2 response was an increase in the total number of Lin^−^Thy1.2^+^ ILCs and KLRG1^+^ ILC2s in the LP (Figure 1b) and MLN (Figure 1c), with increased mucus production in the local tissue (Figure 1d). KLRG1^+^ ILC2s expressed mRNA and protein for *Gata3*, canonical type-2 cytokines *Il5* and *Il13* (Figure 1e,f and Supplementary Figure 1A online), high levels of CD25 (Supplementary Figure 1B), and were morphologically similar to purified *Il4*-GFP^+^ CD4^+^ T_H_2 cells (Figure 1g). ILC2s did not express mRNA or protein for *Rorct* (Figure 1e and Supplementary Figure 1A) or express *Il17a* or *Il22* (Figure 1e). Thus, KLRG1 expression on Lin^−^Thy1.2^+^ cells faithfully marked GATA-3^+^ RORγT^−^ cells (Supplementary Figure 1A,C) as reported previously,^20^ and GATA-3^+^ RORγT^−^ cells were significantly expanded in the MLN at day 5 postinfection (Supplementary Figure 1D). IL-4- (Figure 1h) and IL-13-secreting (Figure 1i) CD4^+^ T_H_2 cells were also significantly increased as early as day 5 postinfection and further increased at days 7 and 10 postinfection. Taken together, these data highlight a concomitant innate and adaptive type-2 response developing early following *H. polygyrus* infection.

### *H. polygyrus*-elicited ILC2s secrete IL-2 and IL-4, supporting T_H_2 cell differentiation *in vitro*

**Figure 2:**
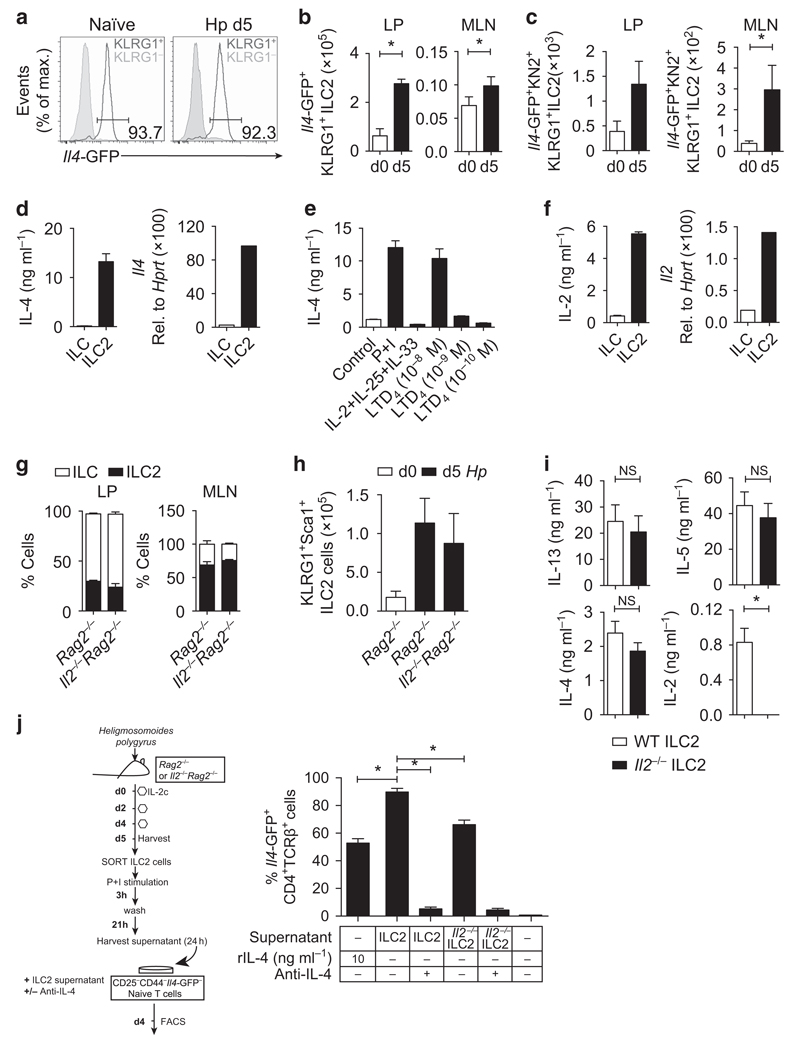
Group-2 innate lymphoid cells (ILC2s) express *Il4*-GFP, secrete interleukin-4 (IL-4), and drive the differentiation of type 2 helper (T_H_2) cells *in vitro* in an IL-4-dependent manner. *Il4*-GFP*Foxp3*-RFP mice were infected with 200 *Heligmosomoides polygyrus* larvae and harvested at day 5 postinfection. (**a**) Representative fluorescence-activated cell sorting (FACS) plots of lamina propria (LP) *Il4*-GFP^+^ cells within KLRG1^−^ (gray line) or KLRG1^+^ (red line) ILC populations. (**b**) Total number of *Il4*-GFP^+^ KLRG1^+^ cells in the LP and mesenteric lymph node (MLN). LP data are representative of three independent experiments with four mice per group. MLN data is pooled from three independent experiments with four mice per group. *Il4*-GFP/KN2 mice were infected with 200 *H. polygyrus* larvae and harvested at day 5 postinfection. (**c**) Total number of *Il4*-GFP^+^ KN2^+^ KLRG1^+^ cells within the Lin^−^Thy1.2^+^ gate. Data are representative of two independent experiments with six mice per group. *Rag2*^−/−^ mice were infected with 200 *H. polygyrus* larvae and harvested at day 5 postinfection. (**d**–**e**) IL-4 or (**f**) IL-2 secretion by purified ILCs and ILC2s restimulated with phorbol 12-myristate 13-acetate and ionomycin (P + I), IL-2 + IL-25 + IL-33, or leukotriene D4 (LTD_4_) for 24 h. (**d**) *Il4* and (**f**) *Il2* gene expression in purified ILCs and ILC2s expressed relative to *Hprt*. Cells were pooled from four to six mice. (**g**) Frequency of ILCs (white bars; Lin^−^Thy1.2^+^ KLRG1^−^) and ILC2s (black bars; Lin^−^Thy1.2^+^ KLRG1^+^) in the LP and MLN of naïve *Rag2*^−/−^ and *Il2*^−/−^*Rag2*^−/−^ mice. *Rag2*^−/−^ or *Il2*^−/−^*Rag2*^−/−^ mice were infected with 200 *H. polygyrus* larvae and harvested at day 5 postinfection. (**h**) Total number of ILC2s (Lin^−^Thy1.2^+^ KLRG1^+^) in the MLN of naïve *Rag2*^−/−^ (white bars) and infected *Rag2*^−/−^ and *Il2*^−/−^*Rag2*^−/−^ mice (black bars). Data are representative of three independent experiments with three to five mice per group. ILC2s were expanded using IL-2 complex (IL-2c) and purified from the MLN of *Rag2*^−/−^ or *Il2*^−/−^*Rag2*^−/−^ mice. Purified ILC2s were stimulated with PMA + I for 3 h, washed, and then plated in complete media for an additional 21 h. (**i**) IL-13, IL-5, IL-4, and IL-2 protein production in the supernatant of restimulated ILC2s (3–24 h). Data are pooled from two independent experiments with three biological replicates per group. (**j**) Model of experimental setup. Frequency of CD4^+^ TCRβ^+^*Il4*-GFP^+^ cells following culture with the supernatant of restimulated ILC2s or *Il2*^−/−^ ILC2s, in the presence or absence of anti-IL-4-blocking antibody. Data are representative of three independent experiments. *P≤0.05 using the Mann–Whitney test. NS, nonsignificant; TCR, T-cell receptor.

We observed that the majority of KLRG1^+^ ILC2s in the LP (Figure 2a) and MLN (Supplementary Figure 2A) of naïve and infected mice were transcribing *Il4*-GFP, in contrast to lung-resident ILC2s and bone marrow (BM) ILC2 progenitors, where only ∼25 and 60% of ILC2s were *Il4*-GFP^+^ (Supplementary Figure 2A). The number of *Il4*-GFP^+^ ILC2s were significantly expanded following *H. polygyrus* infection (Figure 2b), in accordance with observed increases in KLRG1^+^ cells. *Il4*-GFP expression does not always correlate with IL-4 protein secretion.^7^ To determine whether ILC2s were secreting IL-4 *in vivo*, we infected *IL4*-GFP/KN2 mice, which express human CD2 on the surface of cells that have translated and secreted IL-4, and analyzed KN2 expression following infection. Both the proportion (Supplementary Figure 2C) and total number (Figure 2c) of *Il4*-GFP^+^ KN2^+^ cells were increased 5 days following infection. Furthermore, LP ILC2s secreted large amounts of IL-4 protein following *ex vivo* stimulation with phorbol 12-myristate 13-acetate (PMA) and ionomycin (Figure 2d). Although basophils have been described as a dominant *Il4*-GFP^+^ population early in the tissue during *N. brasiliensis* infection,^5^ following *H. polygyrus* infection we observed significantly more *Il4*-GFP^+^ ILC2s in the LP compared with *Il4*-GFP^+^ basophils (Supplementary Figure 2B). To identify factors required for IL-4 secretion from *H. polygyrus*-elicited ILC2s, we stimulated purified ILC2s with cytokines and lipid mediators known to activate ILC2s. Stimulation with IL-2, IL-33, and IL-25 did not induce IL-4 secretion (Figure 2e); however, stimulation with leukotriene D4 (LTD_4_) induced significant IL-4 secretion (Figure 2e), as reported previously,^17^ but did not stimulate IL-5 and IL-13 secretion (Supplementary Figure 3A). Previous reports identified that ILC2s also secrete or express IL-2;^12^,^20^ however, it remains unclear whether ILC2-derived IL-2 is required for ILC2 function. We FACS-purified *H. polygyrus*-elicited KLRG1^+^ ILC2s and confirmed that ILC2s transcribed *Il2* and secreted large amounts of IL-2 protein following PMA and ionomycin stimulation (Figure 2f). Stimulation with IL-25/IL-33 or LTD_4_ did not induce IL-2 secretion from ILC2s (Supplementary Figure 3A), suggesting that other, as yet unknown, factors may stimulate IL-2 secretion from ILC2s *in vivo*. To test the requirements of IL-2 for ILC2 development and IL-4 production, we infected *Il2*^−/−^ mice, which were also backcrossed onto a *Rag2*^−/−^ background, with *H. polygyrus*. In both the LP and the MLN of naïve mice, the frequency and total number of ILC populations were comparable between *Il2*^−/−^*Rag2*^−/−^ and *Rag2*^–/–^ mice (Figure 2g). Similarly, following *H. polygyrus* infection, IL-2 was not required for the expansion of KLRG1^+^ ILC2s in the MLN (Figure 2h). Thus, unlike IL-7, which is essential for the development of all ILC populations,^9^ IL-2 appears to be redundant for ILC development and ILC2 expansion, 5 days following *H. polygyrus* infection. Stimulation of FACS-purified ILC2s with IL-2 alone was insufficient for IL-4 secretion (Supplementary Figure 3B); however, to determine whether IL-2 was required for IL-4 secretion, we isolated ILC2s from the *H. polygyrus*-infected *Il2*^−/−^*Rag2*^−/−^ and *Rag2*^−/−^ mice and restimulated these cells *ex vivo*. IL-2 deficiency had no significant impact on IL-4, IL-13, or IL-5 secretion from ILC2s (Figure 2i), suggesting that IL-2 signaling is not fundamentally required for type-2 cytokine production by ILC2s.

ILC2s can directly^11^,^21^ and indirectly^22^ support T_H_2 cell differentiation. Following the observation that *H. polygyrus*-elicited ILC2s secreted both IL-4 (Figure 2d) and IL-2 (Figure 2e), key cytokines for T_H_2 cell differentiation,^23^,^24^ we hypothesized that ILC2s may be important sources of these cytokines for T_H_2 cell differentiation. We first tested whether the supernatant from *H. polygyrus*-elicited ILC2s could support T_H_2 differentiation *in vitro* by culturing naïve CD4^+^ CD25^−^CD44^+^ *Il4*-GFP^−^ T cells with ILC2-derived supernatant (model; Figure 2j). Strikingly, the supernatant from ILC2s, in combination with T-cell receptor (TCR) ligation, potently differentiated naïve T cells into *Il4*-GFP^+^ T_H_2 cells in an IL-4-dependent manner (Figure 2j). Of note, when we cultured naïve *Il4*-GFP^−^ T cells with the supernatant from *Il2*^−/−^ ILC2s, we observed a slightly lower frequency of *Il4*-GFP^+^ T_H_2 cells (Figure 2j). Taken together, these data indicate that ILC2-derived IL-4 can drive T_H_2 differentiation in a contact-independent manner and that IL-2-competent ILC2s are required for optimal ILC2-mediated T_H_2 differentiation *in vitro*. Furthermore, given the nonessential role for basophils in early T_H_2 responses to *H. polygyrus*^8^ and the minimal increase in basophils at day 5, these *in vitro* data suggest that ILC2s may also be an important source of IL-4 for T_H_2 differentiation *in vivo*.^25^

### IL-2-expanded ILC2s provide partial immunity to *H. polygyrus*

**Figure 3:**
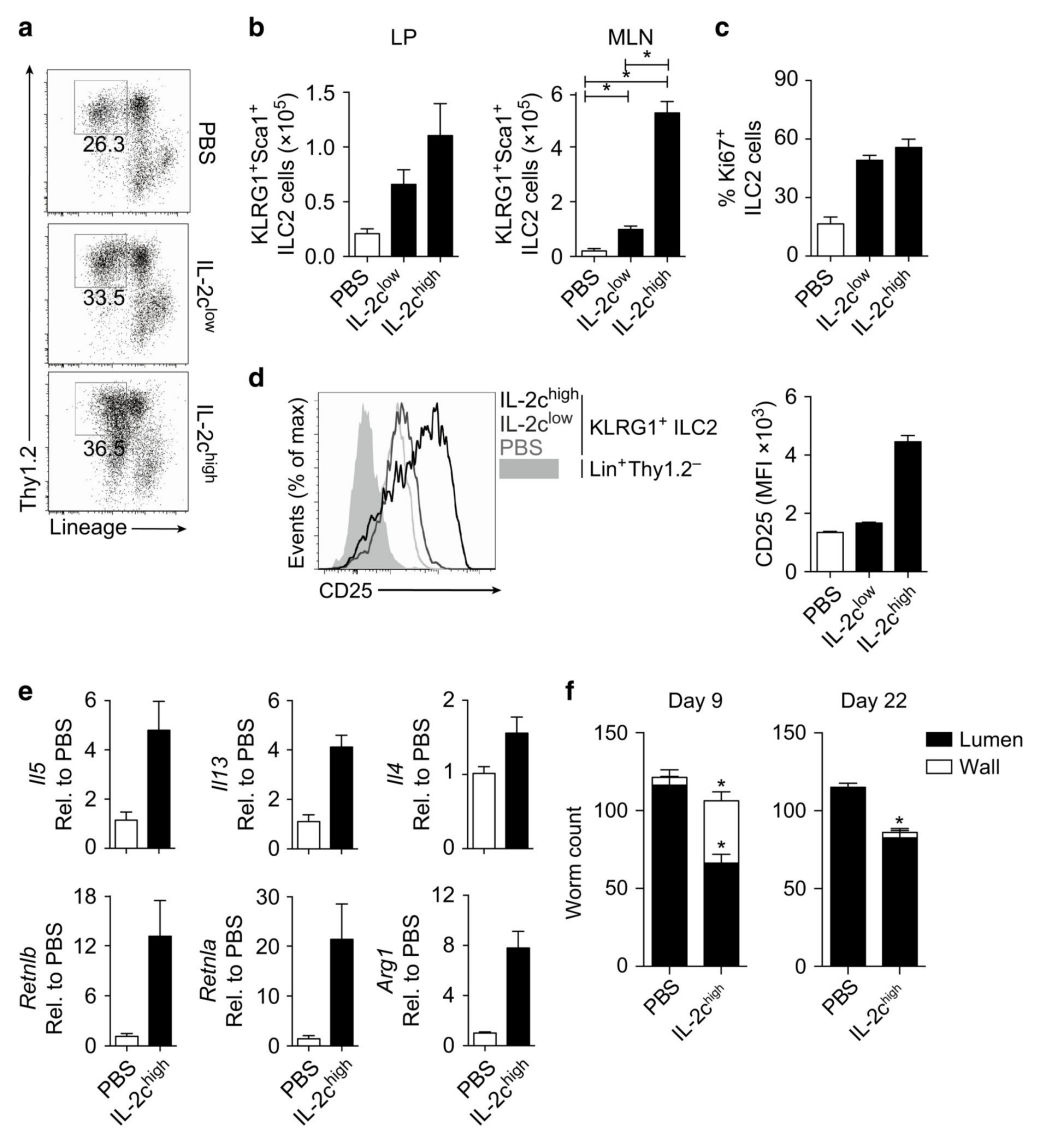
Interleukin (IL-2)-expanded group-2 innate lymphoid cells (ILC2s) can provide functional immunity to *Heligmosomoides polygyrus* infection. *Rag2*^−/−^ were infected with 200 *H. polygyrus* larvae and treated with IL-2 complex (IL-2c) or phosphate-buffered saline (PBS) as a control. Mice were harvested 5 days postinfection. (**a**) Representative fluorescence-activated cell sorting (FACS) plots of mesenteric lymph node (MLN) Lin^−^Thy1.2^+^ cells. (**b**) Total number of ILC2s (Lin^−^Thy1.2^+^ KLRG1^+^) in the lamina propria (LP) and MLN of treated mice. (**c**) Percentage of Ki-67^+^ ILC2s. (**d**) FACS plot and graphical representation of CD25 expression (mean fluorescence intensity (MFI)) on MLN ILC2s (Lin^−^Thy1.2^+^ KLRG1^+^) and Lin^+^ Thy1.2^−^ nonlymphoid cells. (**e**) Gene expression of *Il5*, *Il13*, *Il4*, *Retnla*, *Retnlb*, and *Arg1* in the small intestine (SI) of IL-2c-treated mice, expressed as fold change relative to PBS-treated mice. (**f**) Worm count of trapped larvae in the wall (white bars) and adult worms in the lumen (black bars) of IL-2c-treated mice at days 9 and 22 postinfection. Data are representative of three independent experiments with three to four mice per group. *P≤0.05 using the Mann–Whitney test.

IL-4 is necessary and sufficient to expel *H. polygyrus*^26^,^27^ and *H. polygyrus*-elicited ILC2s produce IL-4 (Figure 2). Following published observations that IL-2:anti-IL-2 cytokine complex (IL-2c) treatment can significantly expand ILC2s *in vivo*,^28^,^29^ we tested whether IL-2c-expanded ILC2s could orchestrate the expulsion of *H. polygyrus* in the absence of CD4^+^ T cells. IL-2c treatment expanded the total number of ILC2s in the LP and MLN at day 5 postinfection (Figure 3a,b). Furthermore, IL-2c treatment increased ILC2 proliferation, determined by Ki67 expression (Figure 3c), and further enhanced CD25 expression on ILC2s (Figure 3d). Expansion of ILC2s correlated with the increased expression of type-2 cytokine genes (*Il5*, *Il13*, and *Il4*) in the SI as well as in increased expression of *Retnla*, *Retnlb*, and *Arg1* (Figure 3e), indicating that downstream type-2 anti-helminth immune-driven pathways were activated. Consequently, we observed more worms trapped in the wall at day 9 that failed to emerge and develop into mature adults, resulting in a significant reduction of adult worms in the lumen at day 22 (Figure 3f), suggesting that IL-2c-expanded ILC2s provided partial protection from *H. polygyrus* infection. However, many worms did emerge and persisted in IL-2c-treated mice, suggesting that despite a fivefold increase in ILC2s (Figure 3b) and the significant activation of type-2 immune pathways, ILC2s alone provided significant, but limited protection from *H. polygyrus* infection.

### ILC2s are required for T_H_2 differentiation *in vivo*, with ILC2-derived IL-4 contributing significantly to ILC2 expansion and T_H_2 differentiation following *H. polygyrus* infection

**Figure 4:**
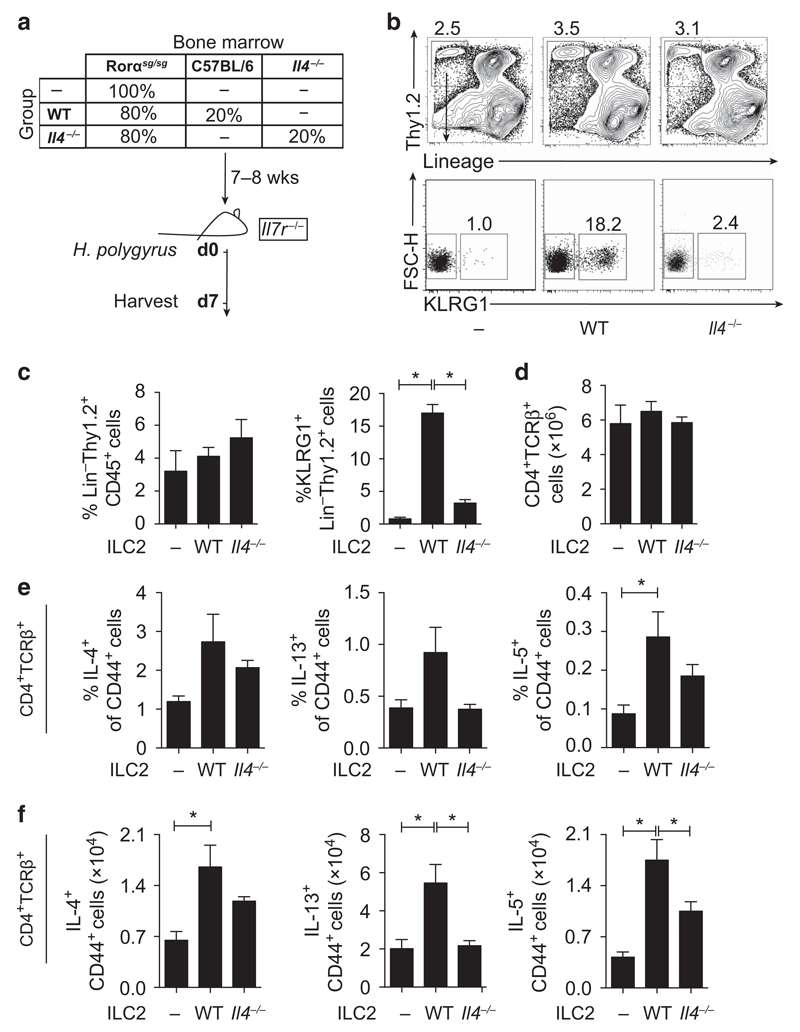
Group-2 innate lymphoid cells (ILC2s) are required for the development of type 2 helper (T_H_2) cells during primary infection with *Heligmosomoides polygyrus*. (**a**) Model of the experimental setup. *Il7r*^–/–^ mice were sublethally irradiated and reconstituted with Rorα^sg/sg^ bone marrow or 80% Rorα^sg/sg^ bone marrow with 20% wild-type (WT) or 20% *Il4*^–/–^ bone marrow. At 7 weeks postreconstitution, chimeric mice were infected with 200 *H. polygyrus* larvae and harvested at day 7 postinfection. (**b**) Representative fluorescence-activated cell sorting (FACS) plots of KLRG1^+^ cells within the Lin^−^Thy1.2^+^ gate and (**c**) frequency of Lin^−^Thy1.2^+^ ILCs and KLRG1^+^ ILC2s in the lamina propria (LP) of Rorα^sg/sg^ (–), Rorα^sg/sg^:WT (WT), or Rorα^sg/sg^:*Il4*^–/–^ (*Il4*^–/–^) mice 7 days postinfection. (**d**) Total number of CD4^+^ TCRβ^+^ cells in the spleen of chimeric mice. (**e**) Frequency and (**f**) total number of cytokine-positive CD4^+^ CD44^+^ cells in the spleen of chimeric mice. Data are representative of two independent experiments with six to seven mice per group. *P≤0.05 using the Mann-Whitney test. FSC-H, forward side scatter of height; Lin, lineage; TCR, T-cell receptor.

The observation that IL-2c-expanded ILC2s could provide significant, but limited, immunity to a primary infection with *H. polygyrus* in the absence of CD4^+^ T cells (Figure 3) was in stark contrast to the ability of ILC2s to drive complete expulsion of *N. brasiliensis* following similar IL-2c treatment.^11^ We therefore explored whether ILC2s could instead be supporting the development of adaptive immune responses *in vivo*, and in particular whether ILC2-derived IL-4 contributed to T_H_2 differentiation *in vivo* following *H. polygyrus* infection. To test the requirement of ILC2s and ILC2-derived IL-4, we reconstituted ILC-deficient mice (*Il7r*^−/−^)^30^ with either ILC2-deficient BM (Rorα^sg/sg^;^31^) alone or in a 80:20 ratio with either *Il4*^−/−^ or WT BM (model; Figure 4a). These chimeric mice allowed us to test whether ILC2s or IL-4 production by ILC2s contributed to the differentiation of T_H_2 cells *in vivo*. Following *H. polygyrus* infection, the proportion of ILCs in the LP did not differ significantly between chimeric mice; however, as expected, *Il7r*^−/−^ mice reconstituted with Rorα^sg/sg^ BM alone (group “–”) were devoid of KLRG1^+^ ILC2s in the LP and MLN, but had normal frequencies of other ILCs (Figure 4b,c and Supplementary Figure 4F). Although IL-4 is not required for ILC2 development,^32^ IL-4 deficiency in ILC2s also impaired their recruitment to the SI and MLN (Figure 4b,c and Supplementary Figure 4A). Total splenic CD4^+^ T cells were comparable between chimeric mice (Figure 4d); however, chimeric mice devoid of ILC2s had lower frequencies and lower total number of IL-4-, IL-13-, and IL-5-secreting CD4^+^ CD44^+^ T cells in the spleen and MLN following *H. polygyrus* infection (Figure 4e,f and Supplementary Figures 4G,H). Furthermore, mice reconstituted with *Il4*^−/−^ BM also displayed fewer IL-4-, IL-13-, and IL-5-secreting CD4^+^ CD44^+^ T cells in the spleen and MLN (Figure 4e,f and Supplementary Figures 4B,C). Whether the reduction of T_H_2 cells in chimeric mice with *Il4*^−/−^ ILC2s was because of fewer ILC2 numbers (Figure 4b,c) is currently unclear. Nevertheless, these *in vitro* and *in vivo* observations clearly identify a significant role for ILC2s in promoting T_H_2 cell differentiation following *H. polygyrus* infection.

### Discussion

The differentiation of T_H_2 cells and subsequent activation of B cells secreting high-affinity immunoglobulin E and immunoglobulin G1 antibodies has been the cornerstone of anti-helminth vaccine efforts. However, the precise mechanisms involved in T_H_2 cell differentiation *in vivo* remain poorly understood. Although a clear role for IL-4R signaling in T cells has been well established *in vitro* and *in vivo*,^33^ the source of IL-4 *in vivo* is still unclear.^25^ The ability of ILC2s to secrete type-2 cytokines^9^,^10^ and present antigen to T cells^11^ has established an important role for ILC2s in initiating type-2 immunity and supporting adaptive immunity. The majority of our current understanding of the role of ILC2s in anti-helminth immunity is derived from murine infections with the rat helminth, *N. brasiliensis*. Although this has been a useful model to study ILC2 biology, whether murine infections with the mouse-adapted *N. brasiliensis* reveal all of the characteristics of ILC2s is unclear. Furthermore, immunity to *N. brasiliensis* does not require IL-4 (ref. 6) and therefore this model system may not be the most appropriate to identify critical sources of IL-4 for anti-helminth immunity. In this study, using the natural murine parasite, *H. polygyrus*, we identified that ILC2s secreted significant amounts of IL-4 *in vivo* and *ex vivo*, which was required to stimulate T_H_2 differentiation *in vitro* and *in vivo*. Restricting *Il4* deficiency to ILC2s *in vivo* revealed an important role of ILC2s for the differentiation of IL-13^+^ and IL-5^+^ T_H_2 cells following infection with *H. polygyrus*.

Eosinophils and basophils have also been reported to secrete IL-4 following *H. polygyrus* infection.^7^ Although both of these sources of IL-4 are redundant for T_H_2 differentiation during *H. polygyrus* infection,^2^,^8^ the interplay between these three innate cell populations (basophils, ILCs, and eosinophils) has recently been reported. Specifically, basophil-derived IL-4 was required to activate ILC2s^34^ and ILC2-derived IL-5 was sufficient to support eosinophilia.^12^ Thus, these three innate cell populations can establish an early type-2 inflammatory landscape, activating innate and adaptive cells and reorganizing local tissue. Whether such interactions are required for optimal IL-4 secretion from ILC2s during *H. polygyrus* is unclear.

In this study, we identified that LTD_4_, but not IL-2, IL-25, or IL-33, stimulated IL-4 secretion by ILC2s. Instead, IL-2, IL-25, and IL-33 stimulated IL-5 and IL-13 secretion, as reported by many others.^9^,^10^ These results suggest that different activating signals stimulate differential secretion of cytokines from ILC2s. In particular, IL-4 secretion appears to be uncoupled from IL-5 and IL-13 secretion in ILC2s. Mast cells, which are highly responsive to tissue damage-associated IL-33^35^ and form close associations with ILC2s *in vivo*,^29^ are an important source of LTD_4_.^36^ Following *H. polygyrus* infection, early activation of mast cells^37^ was required for T_H_2 differentiation,^37^ although the precise mechanisms were unclear. From these reports and our results here, we propose a model where mast cell-derived LTD_4_, following the secretion of tissue damage-associated IL-33, stimulates IL-4 secretion by ILC2s, supporting early T_H_2 differentiation. Indeed, using *Il4*-GFP/KN2 reporter mice, we confirmed that ILC2s secreted IL-4 in the MLN and LP, early during *H. polygyrus* infection. Of note, a recent report identified that *Kit*-deficient mice (*Kit^W^/Kit^W-v^*), which are devoid of mature mast cells, were also deficient in ILC2s.^38^ If *Kit*-deficient mice are indeed deficient in ILC2s, then studies investigating the role of mast cells using these mice may need to be readdressed. In addition to LTD_4_, we observed that *Il2*^−/−^ILC2s secreted slightly less IL-4 and were not as efficient at driving T_H_2 differentiation *in vitro*. These subtle defects may be because of the absence of an IL-2 autocrine feedback loop supporting IL-4 production, given that ILC2s express high levels of the high-affinity IL-2Rα (CD25)^9^ and are highly responsive to IL-2 *in vitro*^9^ and *in vivo*.^39^ A similar IL-2 autocrine loop supporting IL-4 production has been reported in T cells following TCR engagement.^23^ Alternatively, ILC2-derived IL-2 may facilitate T_H_2 differentiation by providing IL-2 directly to T cells, as IL-2 signaling in T cells is required for optimal T_H_2 differentiation.^40^ Finally, IL-4 can also activate ILC2s,^34^ providing an additional feedback loop, with ILC2-derived IL-4 facilitating local ILC2 activation.

During *H. polygyrus* infection, we observed a significant increase in IL-4^+^ ILC2s in the tissue (Figure 2). In addition to the requirement of ILC2-derived IL-4 for T_H_2 differentiation, whether ILC2-derived IL-4 activates other innate, adaptive, or stromal cells is unclear. Indeed, IL-4R-mediated activation of tissue macrophages^41^ and epithelial cells^42^ is required for control of *H. polygyrus*. It has also been reported that IL-4R signaling in dendritic cells is required for optimal T-cell differentiation *in vivo*.^43^ Furthermore, ILC2s can activate macrophages *in vitro*^44^ and support epithelial cell repair in the airways.^14^ We found that IL-2c-mediated expansion of ILC2s in the absence of adaptive immunity led to a significant increase in epithelial cell-associated *Retnlb*^27^ and macrophage-associated *Arg1* expression (Figure 3), suggesting that ILC2-derived IL-4 or IL-13 may have a role in activating these anti-helminth pathways. It has previously been reported that IL-1β may impede ILC2 expansion in WT mice by regulating IL-25 and IL-33 secretion following *H. polygyrus* infection.^3^ In this study, we report that expansion of ILC2s following IL-2c treatment correlated with a reduction in worm establishment, indicating that ILC2s can support immunity following *H. polygyrus* infection. Indeed, ILC2 frequency was shown to correlate with increased type-2 immunity and resistance to *H. polygyrus* in genetically resistant mice.^19^

In conclusion, using a natural murine helminth infection system to model chronic human helminth infection, we have identified an important and previously unappreciated role for ILC2-derived IL-4 for T_H_2 cell differentiation *in vitro* and *in vivo*. It was recently reported that helminth-infected children had reduced ILC2s,^45^ and given that human ILC2s also secrete IL-4,^46^ strategies aimed at enhancing ILC2 function may increase the efficacy of anti-helminth vaccine approaches by boosting T_H_2 cell responses and type-2 immunity.

## Methods

### Animals and generation of BM chimeric mice

All mice used in this study were maintained under specific pathogen-free conditions at the Mill Hill Laboratory, The Francis Crick Institute (London, UK). C57BL/6, *Rag2*^–/–^, *Il2*^–/–^*Rag2*^–/–^, and TRE-IL-7R.B IL-7RecKOrtTA.C (*Il7r*^–/–^) mice were bred and maintained at The Francis Crick Institute. *Il4*-GFP*Foxp3*-RFP mice were generated by crossing 4get^7^ and FIR^47^ mice at The Francis Crick Institute. C57BL/6 *Il4*^–/–^ BM was kindly provided by Judi E Allen (University of Edinburgh, Edinburgh, UK). Rorα^sg/sg^ mice were obtained from Jackson Labs (Farmington, CT) and KN2 (ref. 7) mice were provided by Andrew MacDonald (University of Manchester, Manchester, UK) and crossed with 4get^7^ mice at The Francis Crick Institute. BM cells were isolated either by flushing adult bones or gently crushing bones from neonatal mice using a pestle and mortar. BM cells were filtered through a 40 μm filter, mixed at the required ratios as indicated, and diluted in sterile phosphate-buffered saline (PBS) for intravenous delivery. A total of 3–5 × 10^6^ cells were transferred per mouse and left to reconstitute for 7–8 weeks before the start of the experiment. Animal experiments were performed according to institutional guidelines and following UK Home Office regulations (project license 80/2506) and were approved by The Francis Crick Institute Ethical Review Panel.

### *H. polygyrus* infection and *in vivo* treatments

Mice were infected with 200 *H. polygyrus* L3 larvae by oral gavage. Worms were counted in the lumen or wall of the intestine at days 9 and 22 following infection. For the expansion of ILCs, *Rag2*^–/–^ were treated with IL-2 complex (IL-2c) formed of recombinant IL-2 (R&D, Abingdon, UK) and anti-IL-2 antibody (clone JES6-1A12; BioXcell, West Lebanon, NH). They were prepared at a 1:10 ratio of IL-2:anti-IL-2 in sterile PBS for intraperitoneal delivery. Two doses were used in this study, IL-2c^low^ (0.5 μg:5 μg) and IL-2c^high^ (2.5 μg:25 μg). Mice were given three intraperitoneal doses of IL-2c on days 0, 2, and 4 (as specified in figure legends).

### Cell isolation, RNA extraction, and quantitative real-time polymerase chain reaction

MLN cells were made into a single-cell suspensions by gently mashing through a 40 μm filter (Thermo-Scientific, Loughborough, UK) and prepared for FACS analysis or sorting. For the isolation of LP cells, Peyer’s patches and adipose tissues were removed from the SI, dissected longitudinally to remove faecal contents, and cut into ∼2-in segments. Mucus was scraped off before resuspension in PBS containing 5% fetal bovine serum (FBS) and 25 mm HEPES (Lonza, Slough, UK). The IEL fraction was removed by incubating the intestines in PBS containing 10% FBS, 15 mm HEPES, 5 mm EDTA (Life Technologies, Paisley, UK), and 1 mm dithiothreitol (Sigma, Gillingham, UK) for 25 min at 37 °C. IEL fractions were discarded through a wide mesh into a beaker. The remaining LP was incubated in cIMDM (complete Iscove’s modified Dulbecco’s medium (Gibco, Loughborough, UK) containing 10% FBS, 1 mm EDTA, 100 U ml^−1^ penicillin and 100 μg ml^−1^ streptomycin (Gibco), 8 mm l-glutamine (Gibco), and 0.05 mm 2-mercaptoethanol (Gibco)) containing 0.5 mg ml^−1^ Liberase TL (Roche, Burgess Hill, UK) and 60 μg ml^−1^ DNAse (Roche) for 25–30 min in a 37 °C shaker. Cells were layered onto 40% isotonic Percoll (GE Healthcare, Little Chalfont, UK) to recover leukocytes from digested tissue. Cells were resuspended in cIMDM and prepared for FACS analysis or sorting. For cytospins, 200 000 purified cells were fixed onto a slide and stained with a modified Giemsa stain (Sigma). Cells were identified based on cell morphology and staining characteristics. Slides were scanned (60X/1.4 magnification) using an Olympus IX71 inverted microscope (Olympus, Southend, UK), captured with a Camera QIClick colour CCD camera (QImaging, Surrey, BC, Canada) and processed using Image Pro-Plus software (Media Cybernetics, Marlow, UK). and images were analyzed using Fiji (PMID 22743772) and Photoshop (Adobe, Maidenhead, UK). For quantitative real-time polymerase chain reaction, tissue samples were harvested into RNA*later* (Life Technologies). Tissue samples were homogenized in 500 μl Qiazol using a Precellys Homogenizer (Precellys, Stretton, UK). Two hundred microliters of chloroform was added and the samples were shaken and left at room temperature for 10 min. The samples were spun in a table-top microcentrifuge at a maximum speed for 15 min. Supernatants were added to EtOH and samples were processed using the RNAeasy Kit (Qiagen, Manchester, UK) according to the manufacturer’s instructions and eluted in 30–50 μl RNAse/DNAse-free water. Sortpurified cells were harvested and stored in RLT lysis buffer (Qiagen) at − 20 °C. RNA extractions were performed as above. For quantitative real-time polymerase chain reaction, RNA concentrations were quantified using a Nanodrop 1000 (Thermo Scientific). 0.1–1 μg of RNA was reverse transcribed using the Qiagen Quantitect Reverse Transcription Kit (Qiagen) according to the manufacturer’s instructions. cDNA was used for real-time PCR analysis using *Power* SYBR Green Master Mix and analyzed using an Applied Biosystems 7900HT Fast Real-Time PCR System (both Applied Biosystems, Loughborough, UK). Gene expression was normalized to the housekeeping gene hypoxanthine-guanine phosphoribosyl transferase and expressed as fold change relative to day 0 or PBS treated for some experiments (as detailed in the figure legends). Sequences for primers used are listed in Table 1.

### Flow cytometry and FACS

Cell sorting was performed using a FACS Aria II (BD Biosciences, Oxford, UK). When sorting ILCs from the MLN or LP, single-cell suspensions were obtained as described above, centrifuged (1,500 r.p.m. for 5 min) and stained with antibodies made up in PBS containing 2% FBS. Cells were stained in antibody mix for 25 min at 4 °C, washed, centrifuged (1,500 r.p.m. for 5 min), and resuspended in phenol-red free cIMDM (Gibco; containing 1% 100 U ml^−1^ penicillin and 100 μg ml^−1^ streptomycin (Gibco), 8 mm l-glutamine (Gibco), and 0.05 mm 2-mercaptoethanol (Gibco)) containing 1% FBS and 1 mm EDTA for sorting. Purified fractions were collected into phenol-red free cIMDM containing 20% FBS. FACS analysis was performed using an LSR II (BD Biosciences) analyzer. For FACS analysis, single-cell suspensions were obtained as described above, centrifuged (1,500 r.p.m. for 5 min) and resuspended in cIMDM. A total of 2–6 x 10^6^ cells were stained in 100 μl of antibodies made up in PBS containing 2% FBS for 25 min at 4 °C, washed, centrifuged (1,500 r.p.m. for 5 min), and resuspended in PBS containing 2% FBS. Cells were sometimes fixed in 2–4% paraformaldehyde for 20 min at 4 °C for FACS analysis. For all FACS sorting and analysis, viability of the cells was determined using the LIVE/DEAD Fixable Blue Kit (Life Technologies). Antibodies used include: CD4 (RM4-5: PB (eBioscience, Hatfield, UK), APC (BioLegend, London, UK) and MCD0430: Pacific Orange (Invitrogen, Paisley, UK)), CD25 (PC61: APCCy7 (BioLegend), PerCPCy5.5 (eBioscience)), CD44 (IM7: PeCy7 (BioLegend), PERCPCy5.5 (eBioscience)), CD45 (30-F11: APCCy7 (BioLegend), FITC (eBioscience)), CD11c (N418; APC (BioLegend)), CD11b (M1/70; APC (BioLegend)), CD3 (145-2C11; APC (BioLegend)), TCRγδ (GL3; APC (BioLegend)), TCRαβ (H57-597: APC (eBioscience), PeCy7 and PerCPCy5.5 (BioLegend)), CD19 (6D5; APC (BioLegend)), NK1.1 (PK136; APC (BioLegend)), CD8 (53-6.7; APC (BioLegend)), Ter119 (TER-119; APC (BioLegend)), CD49b (DX5: APC (BioLegend), PB (eBioscience)), Sca1 (E13-161.7: PB (eBioscience)), KLRG1 (2F1: PB (eBioscience)), GATA-3 (L50-823, BV421 (BD Biosciences)), RORγT (Q31-378, PE (BD Pharmingen, Oxford, UK)), IL-5 (554396: APC (BD Biosciences)), IL-13 (eBio13A, FITC (eBioscience)), and huCD2 (S5.5, R-PE (Life Technologies)). ILCs were FACS purified using the following sorting strategy: Live, lymphocytes, CD45^+^, Lineage^−^ (CD3, CD4, CD8, CD19, CD11c, CD11b, NK1.1, TCRβb, TCRγδ, Gr-1, CD49b, Ter119), Thy1.2^+^, KLRG1^+^, and Sca1^+^.

### Cytokine measurements in supernatant

Cytokine concentrations were measured in cell culture supernatants using either FlowCytomix (eBioscience) or LegendPlex Mouse Th1/Th2 Panel (BioLegend) flow cytometry multianalyte detection system for IL-4, IL-2, IL-5, and IL-13 as per the manufacturer’s instructions.

### T-cell differentiation assay

Lin^−^Thy1.2^+^ KLRG1^+^ Sca1 + ILC2s were sort purified from the LP or MLN as described above. Cells were counted, centrifuged (1,500 r.p.m. for 5 min), and resuspended at a final concentration of 5 × 10^4^ cells per 50 μl depending on the experiment. Cells were cultured in cIMDM containing 0.05 mg ml^−1^ PMA (Promega, Southampton, UK) and 0.1 mg ml^−1^ ionomycin (Sigma) for 24 h. For some experiments, cultures were centrifuged after 3 h and the supernatant containing PMA and ionomycin was replaced with fresh cIMDM for the remaining 21 h. Supernatants were harvested 24 h poststimulation and stored at − 20 °C. Naïve T cells were FACS purified as CD4^+^ TCRβ^+^ *Foxp3*-RFP^−^*Il4*-GFP^−^CD25^−^CD44^low^ cells from naïve reporter mice. FACS-purified cells were resuspended in cIMDM at a concentration of 1 × 10^6^/ml. A total of 1 × 10^5^ naïve T cells were plated onto tissue-culture-treated flat-bottom 96-well plates coated with CD3 (1 μg ml^−1^) and CD28 (10 μg ml^−1^) antibody at 37 °C for 2–3 h. Cells were pelleted and resuspended in 50 μl ILC2-derived supernatant with and without the addition of 10 μg ml^−1^ anti-IL-4 antibody (BioXcell).

### Statistical analysis

Data sets were compared by Mann–Whitney test using GraphPad Prism 5 (LaJolla, CA). Differences were considered significant at *P*≤0.05.

## Supplementary Material

Supp Figs

Supp Info

## Figures and Tables

**Table 1:** List of the oligonucleotide primer sequences used

	Forward (5′–3′)	Reverse (5′–3′)
*Hprt*	GCCCTTGACTATAATGAGTACTTCAGG	TTCAACTTGCGCTCATCTTAGG
*Il4*	ACGAGGTCACAGGAGAAGGGA	AGCCCTACAGACGAGCTCACTC
*Il5*	TGACAAGCAATGAGACGATGAGG	ACCCCCACGGACAGTTTGATTC
*Il13*	CCTCTGACCCTTAAGGAGCTTAT	CGTTGCACAGGGGAGTCTT
*Arg1*	GGAAAGCCAATGAAGAGCTG	GCTTCCAACTGCCAGACTGT
*Ym1*	CATGAGCAAGACTTGCGTGAC	GGTCCAAACTTCCATCCTCCA
*Retnla*	CCCTCCACTGTAACGAAGACTC	CACACCCAGTAGCAGTCATCC
*Retnlb*	ATGGGTGTCACTGGATGTGCTT	AGCACTGGCAGTGGCAAGTA
*Il22*	GTGAGAAGCTAACGTCCATC	GTCTACCTCTGGTCTCATGG
*Il17A*	ACCCTGGACTCTCCACCGCAA	GGCTGCCTGGCGGACAATCG
*Rorct*	GGAGCTCTGCCAGAATGACC	CAAGGCTCGAAACAGCTCCAC
*Gata3*	CGGGTCGGCCAGGCAAGATG	AGGGGAXCCTCCCAGCAGGC
*Il2*	CTAGGCCACAGAATTGAAAGATCT	GTAGGTGGAAATTCTAGCATCATCC
