# Supplementary figures and images for: IL-4-producing ILC2s are required for the differentiation of TH2 cells following Heligmosomoides polygyrus infection
Source: Mucosal Immunol. Author manuscript; Available in PMC 2017 Jan 23. (PMC5257265; doi:10.1038/mi.2016.4)

Supplementary Figure 1

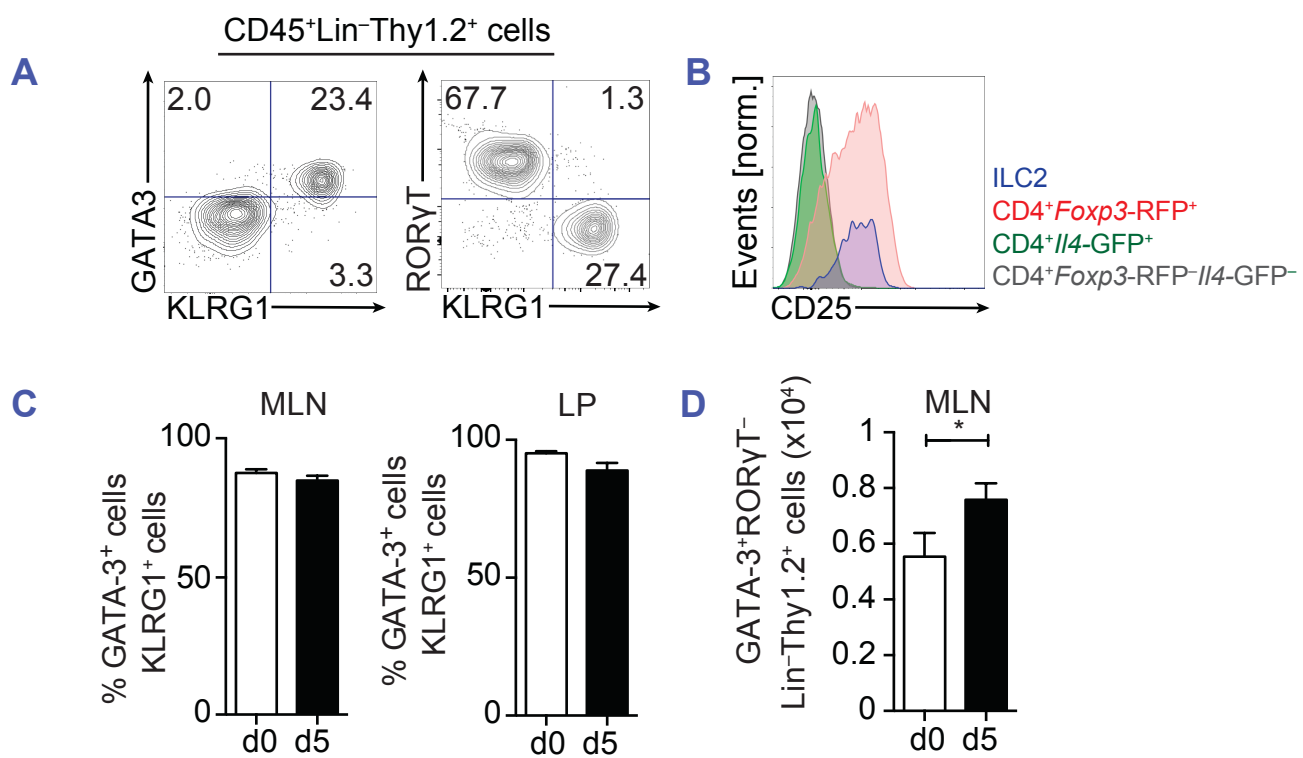

Supplementary Figure 2

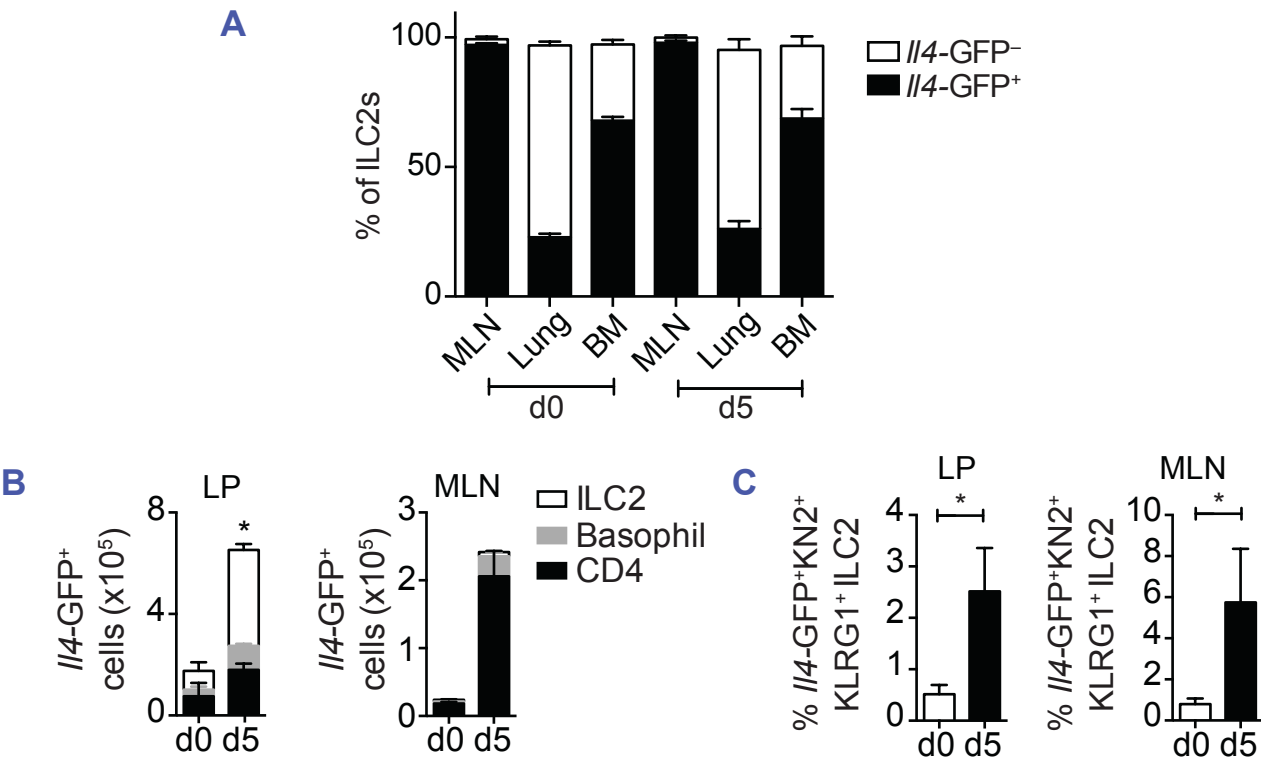

Supplementary Figure 3

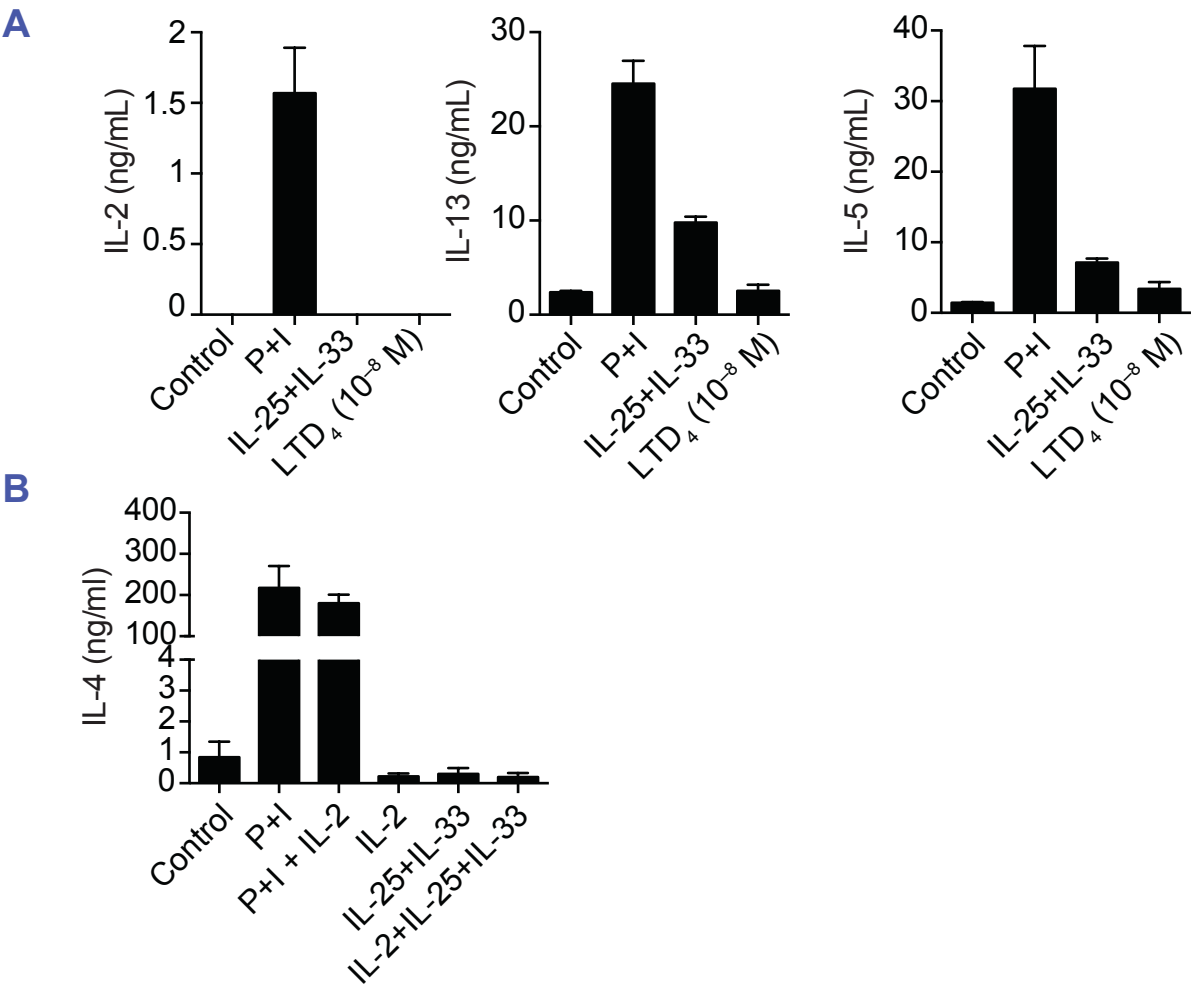

## Supplementary Figure 4

**A**

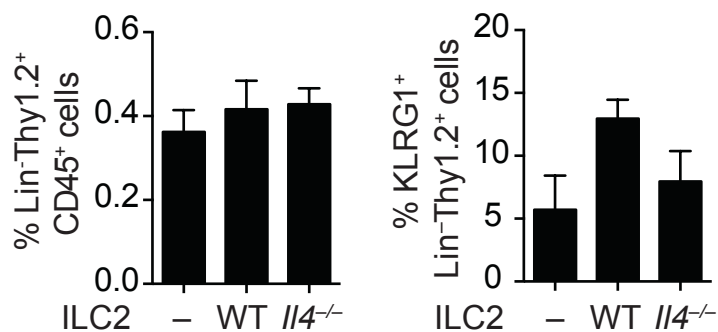

**B**

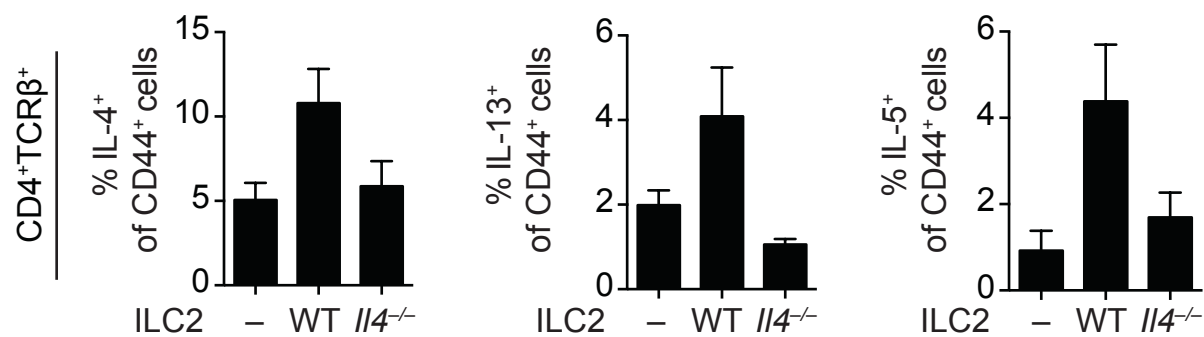

**C**

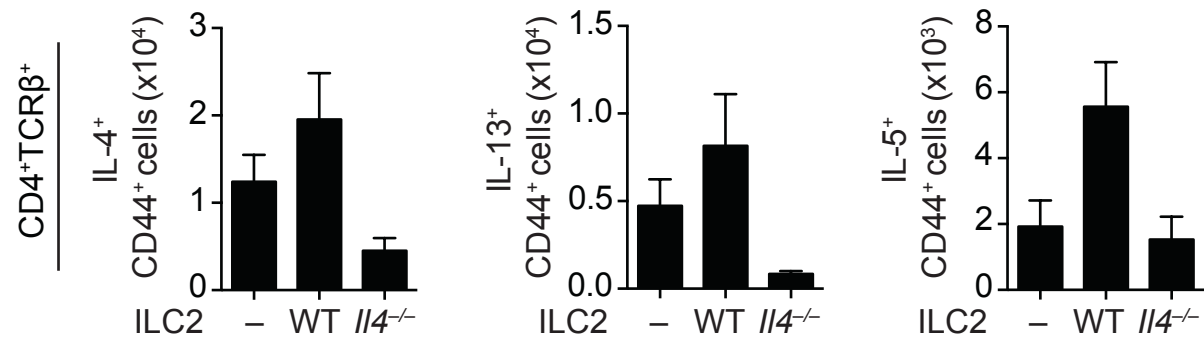

Supplement: Supp Figs [file NIHMS71055-supplement-Supp_Figs.pdf]
