# Supplementary material for: IL-4-producing ILC2s are required for the differentiation of TH2 cells following Heligmosomoides polygyrus infection
Source: Mucosal Immunol. Author manuscript; Available in PMC 2017 Jan 23. (PMC5257265; doi:10.1038/mi.2016.4)
Supplement: Supp Info [file NIHMS71055-supplement-Supp_Info.docx]

**Sup. Figure 1**

*Il4­-*GFP*Foxp3*-RFP mice were infected with 200 *H. polygyrus* larvae and harvested at day 0 and day 5. A) Representative FACS plots of LP KLRG1^+^GATA-3^+^ cells and RORγT^+^ cells within the CD45^+^Lin^–^Thy1.2^+^ gate. B) Representative FACS plot showing CD25 expression (MFI) on MLN ILC2s (Lin^–^Thy1.2^+^KLRG1^+^), *Foxp3-*RFP^+^ Tregs (red line), *Il4*-GFP^+^ T**_H_**2 cells (green line) and *Il4*-GFP^–^*Foxp3-*RFP^–^effector cells (grey line). Data are representative of 2 independent experiments with 4 mice per group. C57BL/6 mice were infected with 200 *H. polygyrus* larvae and harvested at day 0 and day 5. (C) Frequency of MLN and LP GATA-3^+^KLRG1^+^ ILCs D) and total number of MLN GATA-3^+^KLRG1^+^ ILCs. Data are representative of 2 independent experiments with 4 mice per group. * denotes p≤0.05 using Mann-Whitney test.

**Sup. Figure 2**

*Il4­-*GFP*Foxp3*-RFP mice were infected with 200 *H. polygyrus* larvae and harvested at day 0 and day 5. A) Frequency of *Il4-*GFP^+^ cells within MLN- and lung-derived ILC2s (CD45^+^Lin^–^Thy1.2^+^KLRG1^+^CD25^+^) and BM-derived ILC2Ps (CD45^+^Lin^­–^CD25^+^ Thy1.2^+^) at d0 and d5 post-infection. Data are representative of 2 independent experiments with 4 mice per group. B) Total number of CD4 cells (black bar; CD45^+^*Il4-*GFP^+^CD4^+^), basophils (grey bar; CD45^+^*Il4*-GFP^+^CD49b^+^), and ILC2s (white bar; CD45^+^*Il4-*GFP^+^CD4^–^CD49b^–^Thy1.2^+^KLRG1^+^) in the LP and MLN. Data are representative of 3 independent experiments with 4 mice per group. *Il4-*GFP/KN2 mice were infected with 200 *H. polygyrus* larvae and harvested at day 5 post-infection. C) Frequency of LP and MLN KLRG1^+^*Il4*-GFP^+^KN2^+^ cells within the Lin^–^Thy1.2^+^ gate. Data are representative of 2 independent experiments with 6 mice per group. * denotes p≤0.05 using Mann-Whitney test.

**Sup. Figure 3**

ILC2s were expanded using IL-2c and purified from the MLN of *H. polygyrus-*infected *Rag2^–/–^* mice. Purified ILC2s were stimulated with A) PMA+I, IL-25/IL-33 or LTD_4_ for 24h and levels of IL-2, IL-13 and IL-5 secretion were measured in the supernatant. Alternatively, purified ILC2s were stimulated with B) PMA+I, PMA+I/IL-2, IL-2, IL-25/IL-33 or IL-2/IL-25/IL-33 and levels of IL-4 were measured in the supernatant. Data is representative of 2 independent experiments with 3 biological replicates per group.

**Sup. Figure 4**

*Il7r^–/–^* mice were sub-lethally irradiated and reconstituted with Rorα^sg/sg^ bone marrow or 80% Rorα^sg/sg^ bone marrow with 20% WT or 20% *Il4^–/–^* bone marrow (see model, Figure 4). 7-weeks post-reconstitution, chimeric mice were infected with 200 *H. polygyrus* larvae and harvested at day 7 post-infection. A) Frequency of Lin^–^Thy1.2^+^ ILCs and KLRG1^+^ ILC2s in the MLN of Rorα^sg/sg^ (–), Rorα^sg/sg^:WT (WT) or Rorα^sg/sg^*:Il4*^–/–^ *(Il4^–/–^)* mice day 7 post infection*.* B) Frequency and C) total number of cytokine-positive CD4^+^CD44^+^ cells in the MLN of chimeric mice. Data are representative of 2 independent experiments with 6-7 mice per group.
